# Rapid isolation of *Yr9* via MutIsoSeq and QTL analysis of durable stripe rust resistance in wheat cultivar Xingzi 9104

**DOI:** 10.1007/s44154-025-00226-9

**Published:** 2025-04-30

**Authors:** Yibo Zhang, Shuo Huang, Yuqing Li, Shuaiwei Cao, Hui Ren, Mingjie Xiang, Haitao Dong, Jiangna Han, Ying Zhao, Xiangxue Zhang, Xunying Yuan, Qilin Wang, Yajun Wang, Yi Ouyang, Zujun Yang, Zhensheng Kang, Shengjie Liu, Jianhui Wu, Qingdong Zeng, Dejun Han

**Affiliations:** 1 State Key Laboratory of Crop Stress Biology for Arid Areas, College of Agronomy, Northwest A&F University, Yangling, Shaanxi 712100 People’s Republic of China; 2 CAS Center for Excellence in Molecular Plant Sciences, Institute of Plant Physiology and Ecology, Shanghai, 200032 People’s Republic of China; 3 State Key Laboratory of Crop Stress Biology for Arid Areas, Plant Protection, Northwest A&F University, Yangling, Shaanxi 712100 People’s Republic of China; 4 Center for Informational Biology, School of Life Science and Technology, University of Electronic Science and Technology of China, Chengdu, 611731 People’s Republic of China

**Keywords:** Stripe rust, Durable resistance, QTL analysis, Xingzi 9104, *Yr9*

## Abstract

The fungus *Puccinia striiformis* f. sp. *tritici* (*Pst*) is the causal agent of wheat stripe rust which constitutes a major limitation to wheat production. Cloning and applying disease-resistant genes are considered as an effective solution. Chinese wheat cultivar Xingzi 9104 (XZ9104) has exhibited durable resistance across multiple environments since its release. Through quantitative trait loci (QTL) analysis, eight QTL were found on chromosome arms 1BS, 1BL, 2AL, 2BL, 3BS, 4BL, 5BL and 7BL. *YrXZ* identified as 1RS.1BL translocation conferred race-specific all-stage resistance to *Pst* race CYR23. *QYrxz.nwafu-1BL.6* and *QYrxz.nwafu-3BS.7* were considered as the adult plant resistance genes *Yr29* and *Yr30*, respectively. Notably, *QYrxz.nwafu-2BL.5* accounted for 15.75–47.63% of the phenotypic variation across diverse environments and its pyramiding with *Yr29* and *Yr30* can confer high level of resistance. Other QTL were environment-dependent with minor effects. To clone the above resistance genes, we created a population of over 2,000 M_5_ mutants in XZ9104 using ethylmethane sulfonate (EMS) mutagenesis and screened various types of susceptible mutants. Using the MutIsoseq approach with five mutant lines susceptible to race CYR23, we rapid isolated a candidate gene for *YrXZ* encoding coiled-coil nucleotide-binding site leucine-rich repeat (CC-NBS-LRR) protein. Integrating cytological analysis, gene-based association analysis, transcriptomic profiling and virus-induced gene silencing (VIGS), we confirmed that the causal gene for *YrXZ* was indeed *Yr9.* This study demonstrated that multiple QTL with different effects contributed to the durable resistance in XZ9104. Understanding the molecular mechanisms and pathways involved in plant defense can inform future strategies for deploying resistance gene and engineering of genetic resistance against evolving diseases.

**Supplementary Information:**

The online version contains supplementary material available at 10.1007/s44154-025-00226-9.

## Introduction

Wheat (*Triticum aestivum* L.) is one of the most important food crops, providing 35 –40% of the global population's food supply (Shiferaw et al. 2013). However, food production is significantly challenged by diverse diseases, especially those of fungi pathogens. Among these, wheat stripe rust, caused by *Puccinia striiformis* f. sp. *tritici*, is a particularly troublesome fungal disease, characterized by seasonal infection and rapid spread; Stripe rust is widely distributed by means of air-borne urediniospores, and damaging epidemics occur at high frequencies (Chen et al. 2014). This disease is prevalent globally, with a particularly significant impact in major wheat production countries such as China, the United States, Australia, India, Canada and Russia, posing a severe threat to wheat production. Annually, it typically leads to an average yield loss of 10—20%, but in extreme cases, the yield reduction in individual can reach 100% (Srinivas et al. 2024; Chen 2020; Chen 2005).

The identification and utilization of disease resistance genes to address the threat of stripe rust is a key focus in wheat breeding. To date, 87 resistance genes (designated as *Yr1*-*Yr87*) are officially catalogued, more than 100 resistance genes are temporarily identified, and over 300 QTL have been mapped (Jan et al. 2021; Zhu et al. 2023; Klymiuk et al. 2022; Sharma et al. 2024). These genes are sourced from hexaploid common wheat, cultivated and wild tetraploid and diploid wheat and more distantly related species within the crossing range of wheat. Resistance is often classified into two categories: namely, all-stage resistance (ASR) and adult-plant resistance (APR), based on time of expression, but both categories are affected by factors such as time of observation, temperature, and scorer distinction between arbitrary resistant and susceptible responses. ASR, also known as seedling resistance is often highly effective and easily selected by breeders. In contrast, the APR becomes evident at various times post seedling and is more difficult (or expensive) to select, but is reputed to be more durable (Wang et al. 2023a). The presence of a single APR gene does not confer a sufficient level of efficacy. To maximize the effectiveness, it is recommended to integrate the APR gene with other complementary genes. However, relying on a single ASR resistance gene can lead to natural selection and virulence variation among stripe rust physiological races. Given the continuous evolution of these races, there is an urgent need for in-depth research and expansion of the resistance gene pool.

Currently, only a few of these genes have been cloned, including *Yr5a*/*Yr5b* (Marchal et al. 2018), *Yr7* (Marchal et al. 2018), *Yr10/NAM* (Dibley et al. 2024; Liu et al. 2014), *Yr15* (Klymiuk et al. 2018), *Yr18/Lr34* (Krattinger et al. 2009), *Yr27* (Athiyannan et al. 2022), *Yr36* (Uauy et al. 2005), *Yr46*/*Lr67* (Moore et al. 2015), *Yr84* (Klymiuk et al. 2022), *Yr87* (Sharma et al. 2024), *YrAS2388* (Zhang et al. 2019) and *YrU1* (Wang et al. 2020). Traditional map-based cloning, which is commonly used for gene identification in wheat when the functional annotation and coding sequences of target genes remain unknown. However, obtaining a precise genetic interval is challenging due to the limited specific molecular markers and recombination rates. Related wheat species are rich sources of disease resistance genes for improving bread wheat varieties, but differences in chromosome structure and the introduction of foreign genes during improvement impede recombination. In such cases, alternative cloning methods are needed. Mutants are excellent materials for gene isolation and cloning. Currently, several genes such as *Yr5/YrSP, Yr7, YrNAM, Lr9, Sr22, Sr26, Sr43, Sr45, Sr61, Sr62* have been cloned using mutant-based sequencing approaches (Steuernagel et al. 2016; Zhang et al. 2021; Sánchez-Martín et al. 2016; Yu et al. 2023; Yu et al. 2022; Ni et al. 2023; Wang et al. 2023b). With advancement in sequencing technology, mutant analysis and gene identification methods such as MutRenSeq, MutChromSeq, MutRNA-Seq, sequencing trait-associated mutations (STAM), and MutIsoSeq have merged, and the combination of mutant analysis and sequencing is increasingly being adopted for gene cloning.

Xingzi 9104 is a valuable wheat germplasm in China, with a pedigree comprising S69/8131–1// Bezostaya1. Since its release, it has shown resistance to multiple diseases, including stripe rust. Earlier research suggested that, based on the gene derivation method, XZ9104 possessed a dominant gene conferring resistance throughout the entire growth period and at least one gene for adult plant resistance (Liu et al. 2006). The main objectives of this study were: (1) to map the resistance genes in XZ9104 using the 90 K and 660 K arrays; (2) to clone the ASR gene using MutIsoSeq; and (3) to develop molecular markers for resistance loci/genes.

## Results

### Evaluation of stripe rust resistance

**Fig. 1:**
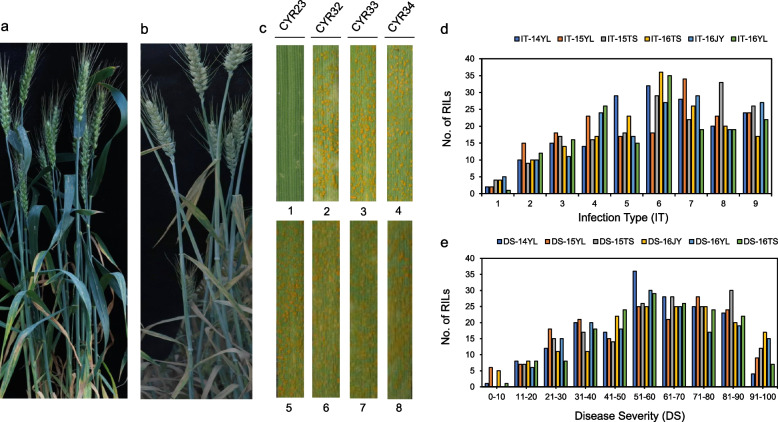
The overall response of XZ9104 (**a**) and AvS (**b**) to stripe rust at adult plant stage, and the typical stripe rust symptoms of wheat lines XZ9104 (c1-4) and AvS (c5-8) after inoculation with different Pst races at seedling stage. **d** Frequency distributions of mean infection type (IT) for 177 RILs from cross AvS × XZ9104 evaluated at Yangling, Jiangyou and Tianshui. **e** Frequency distributions of mean disease severities (DS) for 177 RILs from cross AvS × XZ9104 evaluated at Yangling, Jiangyou and Tianshui

At the two-leaf stage, inoculated with four stripe rust physiological races (CYR23, CYR32, CYR33, CYR34) to assess seedling resistance. XZ9104 was only resistant to CYR23 (IT = 1), whereas the susceptible controls, AvS and MX169, were susceptibility (IT = 7–9) (Fig. 1c). At the heading stage in the field, after inoculation with mixed races, XZ9104 had high resistance to prevalent epidemic races. The results confirmed that XZ9104 displayed a high level of resistance to the prevailing epidemic races (DS = 5–20%), while the susceptible control, such as AvS was highly susceptible (DS = 90–100%) (Fig. 1a, b).

The RIL population inoculated with CYR23 at the seedling stage segregated 87 lines resistant: 90 lines susceptible indicating that resistance was conferred by a gene (Chi^2^_1:1_ = 0.05; *P* = 0.822)(Table S1). The distribution of IT and DS in the field was continuous for all environments indicating quantitative inheritance (Fig. 1d, e). Pearson correlation coefficients in pairwise comparisons across the six environments ranged from 0.86 to 0.92 (*P* < 0.01) (Table 1). Analysis of variance for RILs showed significant phenotypic differences among lines, environments, and line-environment interactions, but not among replicates, with heritability ranging from 0.88 to 0.98, and consistent APR expression across test environments (Table 2).

**Table 1:** Correlation coefficients (r) of stripe rust disease severity (DS) in the AvS × XZ9104 RIL population across field environments

Environment^a^	*r* value based on DS^b^					
	DS-14YL	DS-15YL	DS-15TS	DS-16JY	DS-16YL	DS-16TS
DS-14YL	1.0000^***^	0.8706^***^	0.8646^***^	0.9068^***^	0.9044^***^	0.9004^***^
DS-15YL	0.8706^***^	1.0000^***^	0.8757^***^	0.8712^***^	0.8786^***^	0.8650^***^
DS-15TS	0.8646^***^	0.8757^***^	1.0000^***^	0.8756^***^	0.8626^***^	0.8647^***^
DS-16JY	0.9068^***^	0.8712^***^	0.8756^***^	1.0000^***^	0.9231^***^	0.9063^***^
DS-16YL	0.9044^***^	0.8786^***^	0.8626^***^	0.9231^***^	1.0000^***^	0.9164^***^
DS-16TS	0.9004^***^	0.8650^***^	0.8647^***^	0.9063^***^	0.9164^***^	1.0000^***^

^a^YL, TS, and JY denote Yangling, Tianshui, and Jiangyou, respectively

^b^All r values were significant at *P* = 0.001

**Table 2:** Analysis of variance (ANOVA) for stripe rust and disease severity (DS) data for the AvS × XZ9104 RIL population evaluated at Yangling, Jiangyou and Tianshui in 2015, 2016 and 2017

Source of variation	DS			*P* value
	df	Mean square	F value	
RILs	173	5441.8286	2604.011	< 0.001
Environments	5	870.4996	416.5494	< 0.001
RILs × environment	844	121.9702	58.3649	< 0.001
Error	1017	2.0898		
*h*^2^_b_	0.9781			

### Genetic linkage map

**Fig. 2:**
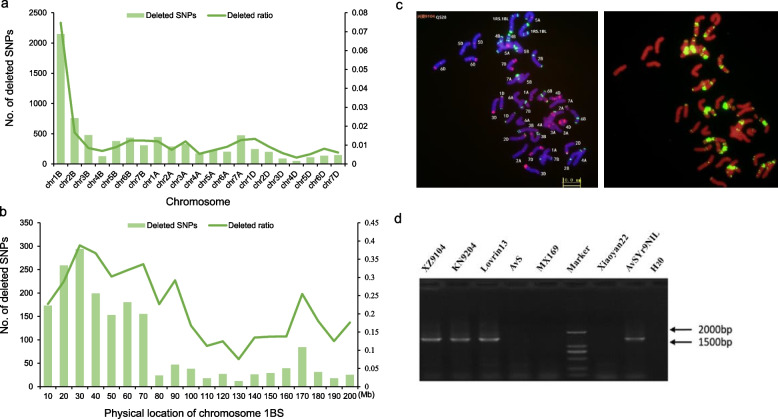
Deletion SNP analysis was performed on the whole genome and 1BS chromosome using a wheat 660 K SNP array (**a**, **b**). **c** The wheat disease-resistant material XZ9104 was identified by FISH. Oligo-pSc119.2 (green) and Oligo-pTa535 (red) were labeled as FISH probes. Scale bars, 10 μm (color figure online). **d** The molecular marker *Yr9 (1B. 1R)-H20* was used to verify the materials involved in this study. AvS, MX169 and XY22 were *Yr9* negative controls

Genotyping of 177 RIL and parents was carried out using the 90 K SNP array. A total of 12,725 SNPs were polymorphic between the parents, and the SNPs with data missing > 10% or partial separation were removed. The linkage map of 21 linkage groups was generated using the remaining 1480 SNPs. The A, B, and D genomes had 660, 625, and 195 SNP markers, respectively. The linkage map spanned 4,054.17 cM with an average bin interval of 3.54 cM (Table S2). Upon removing SNPs with a high deletion rate, more deletions were observed for chromosome arm 1BS. Re-genotyping with the higher density 660 K array data, suggested that the 1BS chromosome of XZ9104 might have undergone structural change or actual deletion, leading to a large number of SNPs deletion of 1BS (Fig. 2a, b).

### Mapping QTL for stripe rust resistance

**Fig. 3:**
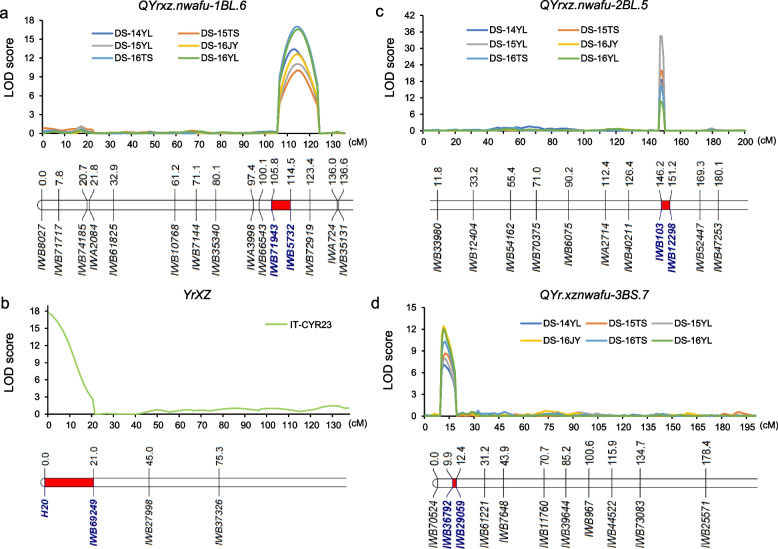
Graphical display of stripe rust QTL mapping regions on chromosome arms 1BL (**a**), 2BL (**c**), 3BS (**d**) in six environments in the field, and 1BS (**b**) in the seeding stage, respectively. The flanking markers colored with blue, and the red bar represents the target interval

Using the complete interval mapping method, three stable QTL which are stable in all test environments for stripe rust resistance from XZ9104 were detected on chromosomes 1B, 2B, and 3B. *QYrxz.nwafu-1BL.6*, *QYrxz.nwafu-2BL.5*, *QYrxz.nwafu-3BS.7* were detected in all environments and thus considered stable. *QYrxz.nwafu-1BL.6* was located between SNP markers *IWB71943* and *IWB5732*, within the 3.55 Mb interval of 667.23—670.78 Mb, explained 10.2—23.49% of the phenotypic variation (Table 3; Fig. 3a). Among these QTL, *QYrxz.nwafu-2BL.5* had the largest effect, explaining 15.75–47.63% of the phenotypic variation in different environments, and was mapped between markers *IWB103* and *IWB12298*, spanning of 4.99 cM (Fig. 3c). *QYrxz.nwafu-3BS.7* could explain 8.45–18.18% of the phenotypic variation was located between markers *IWB36792* and *IWB29059*, with a distance of 0.81 Mb (Table 3; Fig. 3d). Additionally, four QTL with small effect values (< 10%) were identified, named *QYrxz.nwafu-2AL.2*, *QYrxz.nwafu-4BL.4, QYrxz.nwafu-5BL.2, QYrxz.nwafu-7BL.5*, were considered unstable or environmentally-dependent (Table 3).

**Table 3:** Summary of stripe rust resistance QTL detected in the AvS × XZ9104 RIL population in the adult plant stage using IciMapping V4.2

QTL, environment^a^	Flanking marker	Genetic position^b^ (cM)	Physical interval^c^ (Mb)	LOD^d^	PVE^e^	ADD^f^
DS-14YL	IWB71943-IWB5732	113	667.23–670.78	12.1212	16.1722	−9.1703
DS-15YL	IWB71943-IWB5732	114	667.23–670.78	10.2372	10.2354	−7.8685
DS-15TS	IWB71943-IWB5732	114	667.23–670.78	9.2468	11.6761	−7.792
DS-16JY	IWB71943-IWB5732	114	667.23–670.78	9.8755	13.881	−8.9691
DS-16YL	IWB71943-IWB5732	114	667.23–670.78	15.0874	23.4916	−10.4059
DS-16TS	IWB71943-IWB5732	114	667.23–670.78	18.2526	21.9893	−10.2681
*QYrxz.nwafu-2BL.5*						
DS-14YL	IWB103-IWB12298	148	677.22–683.05	18.5715	26.2844	−11.7591
DS-15YL	IWB103-IWB12298	148	677.22–683.05	34.4977	47.6303	−17.2176
DS-15TS	IWB103-IWB12298	148	677.22–683.05	21.8926	33.4064	−13.2901
DS-16JY	IWB103-IWB12298	148	677.22–683.05	15.0146	22.9811	−11.6238
DS-16YL	IWB103-IWB12298	148	677.22–683.05	10.9813	15.7549	−8.6519
DS-16TS	IWB103-IWB12298	148	677.22–683.05	16.0595	19.4287	−9.7355
*QYrxz.nwafu-3BS.7*						
DS-14YL	IWB36792-IWB29059	11	6.25–7.06	8.6968	10.6528	−7.3683
DS-15YL	IWB36792-IWB29059	12	6.25–7.06	8.791	8.4529	−7.0967
DS-15TS	IWB36792-IWB29059	12	6.25–7.06	9.1147	11.2865	−7.6036
DS-16JY	IWB36792-IWB29059	11	6.25–7.06	12.7753	18.1811	−10.1917
DS-16YL	IWB36792-IWB29059	11	6.25–7.06	11.4514	17.1481	−8.8492
DS-16TS	IWB36792-IWB29059	11	6.25–7.06	12.6636	14.0759	−8.1465

^a^YL, JY, and TS, Yangling, Jiangyou, and Tianshui; 14, 15, and 16, growing seasons 2014–2015, 2015–2016, 2016–2017

^b^Peak position in centiMorgans (cM) from the first linked marker in the relevant linkage group

^c^Physical location in mega bases (Mb) from linked markers in the wheat genome

^d^Logarithm of odds score

^e^Percentages of phenotypic variation explained by individual QTL

^f^Additive effect of the allele. Negative sign indicates that the favorable allele is from XZ9104

Based on the resistance spectrum of XZ9104 and the deletion of numerous genotypes, it was hypothesized that XZ9104 carried the 1BL 1RS translocation. To confirm this, the 1RS chromosome-specific molecular marker *H20* was used to genotype the RIL population and its parents and controls. Electrophoresis results showed rye-positive bands, and cytological analysis identified a 1BL 1RS translocation in XZ9104 (Fig. 2c). The *H20* marker exhibited a high co-segregation with resistance to race CYR23. Using QTL IciMapping v4.2, the resistance locus was mapped between the markers *H2O* and *IWB7123*, accounting for 34.96% of the phenotypic variation and the resistance allele was designated *YrXZ* (Table S3; Fig. 3b). Currently, no stripe rust resistance gene other than *Yr9* has been identified within the 1BS (1RS) region, suggesting that the resistance of XZ9104 to CYR23 at the seedling stage is due to the presence of the *Yr9* gene.

### QTL combinations and interaction

**Fig. 4:**
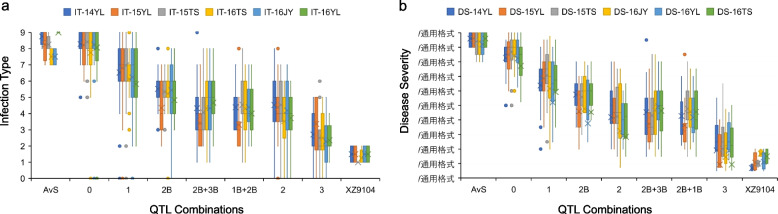
(**a**) Effects of different combinations of quantitative trait loci (QTL) on stripe rust reaction using infection type (IT) data for the AvS × XZ9104 RIL population from Yangling (YL), Tianshui (TS), and Jiangyou (JY). **b** Effects of different combinations of quantitative trait loci (QTL) on stripe rust reaction using disease severity (DS) data for the AvS × XZ9104 RIL population from Yangling (YL), Tianshui (TS), and Jiangyou (JY)

To study the impact of QTL combinations, RILs were categorized into multiple genotype groups across all field experiments based on flanking molecular markers genotypes (Table S4). The results showed a consistent linear increase in disease resistance with the accumulation of QTL. RILs possessing *QYrxz.nwafu-1BL.6, QYrxz.nwafu-2BL.5*, and *QYrxz.nwafu-3BS.7* simultaneously exhibited superior resistance, approaching that of XZ9104. Notably, *QYrxz.nwafu-2BL.5* had the highest individual effect, almost equivalent to the combined effect of the other two QTL. Moreover, when *QYrxz.nwafu-2BL.5* was combined with either *QYrxz.nwafu-1BL.6* or *QYrxz.nwafu-3BS.7*, there was no significant effect in reducing stripe rust severity (Fig. 4a, b), suggesting possible epistatic interaction involving the latter two alleles..

### A gene candidate for *YrXZ*

**Fig. 5:**
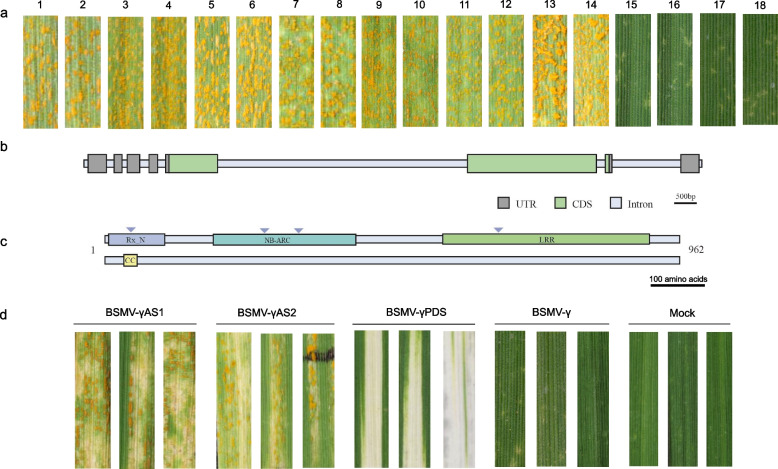
(**a**) The response of the selected EMS susceptible mutants to the race CYR23 of wheat stripe rust. 1–10 were mutation lines, 11–12 were the susceptible parent AvS, 13–14 were the susceptible control MX169, 15–16 were the resistant control KN9204, and 17–18 were the wild type XZ9104. **b** Gene structure of The *YrXZ* (*Yr9*). **c** The predicted protein structure of *YrXZ* and the location of the mutation sites of each mutant (marked by blue inverted triangle). **d** Representative images showing the results of the VIGS experiment using barley stripe mosaic virus (BSMV). BSMV-γas1 and BSMV-γas2 indicate two silencing constructs targeting *YrXZ*, BSMV-γ indicates control, and BSMV-γPDS indicates silencing construct targeting the phytoene desaturase genes. Mock represents the results of wild-type inoculation without silencing any structure

MutIsoSeq was used to clone the *YrXZ* gene. Screening the EMS-induced mutant population in the XZ9104 genetic background identified 5 susceptible mutants. Iso-seq reads were obtained from RNA of XZ9104 leaves inoculated with CYR23, and RNA-seq reads of the susceptible mutants were mapped to Iso-seq reads. Only one point- mutation transcript was obtained among the sequenced mutants (M738, M378, M145, MX990, M823). Mutant M738 had a missense mutation at 872 bp (glutamic acid to alanine), M378 was prematurely terminated at 1,372 bp, M145 and MX990 had a missense mutation at 1,517 bp (threonine to isoleucine), and M823 had a missense mutation at 2,444 bp (glycine to aspartic acid) (Table S5; Fig. 5c).

The putative *YrXZ* encoded a 962 amino acids protein with CC (coiled-coil) N-terminal domain and NB-ARC domain structure (Fig. 5b, c). Blastn analysis showed that *TraesKN1B01HG01710.1* in the 1B.1R translocation line Kenong 9204 genome had 100% similarity to *YrXZ*. In the Chinese Spring reference genome, *TraesCS1A03G0066200.1* and *TraesCS1D03G0054800.1* had the highest identities with *YrXZ* (92% and 91%, respectively). The sequence *SECCE1Rv1G0003760.1* from LO7 (rye) also had 100% identity with *YrXZ*. A virus-induced gene silencing (VIGS) experiment using barley stripe mosaic virus significantly reduced the stripe rust resistance of plants with the target fragment (Fig. 5d; S5a). Moreover, sequence analyses indicated that the germplasms containing *Yr9*, including Lovrin 10, Lovrin 13, and AvS*Yr9*NIL, exhibited 100% similarity to the coding sequences of *YrXZ*. Comprehensive transcriptome analysis, along with mutant analysis, genetic linkage mapping, and transient silencing, indicated that *YrXZ* corresponds to *Yr9*.

### Markers for MAS of *QYrxz.nwafu-2BL.5* and *Yr9*

**Fig. 6:**
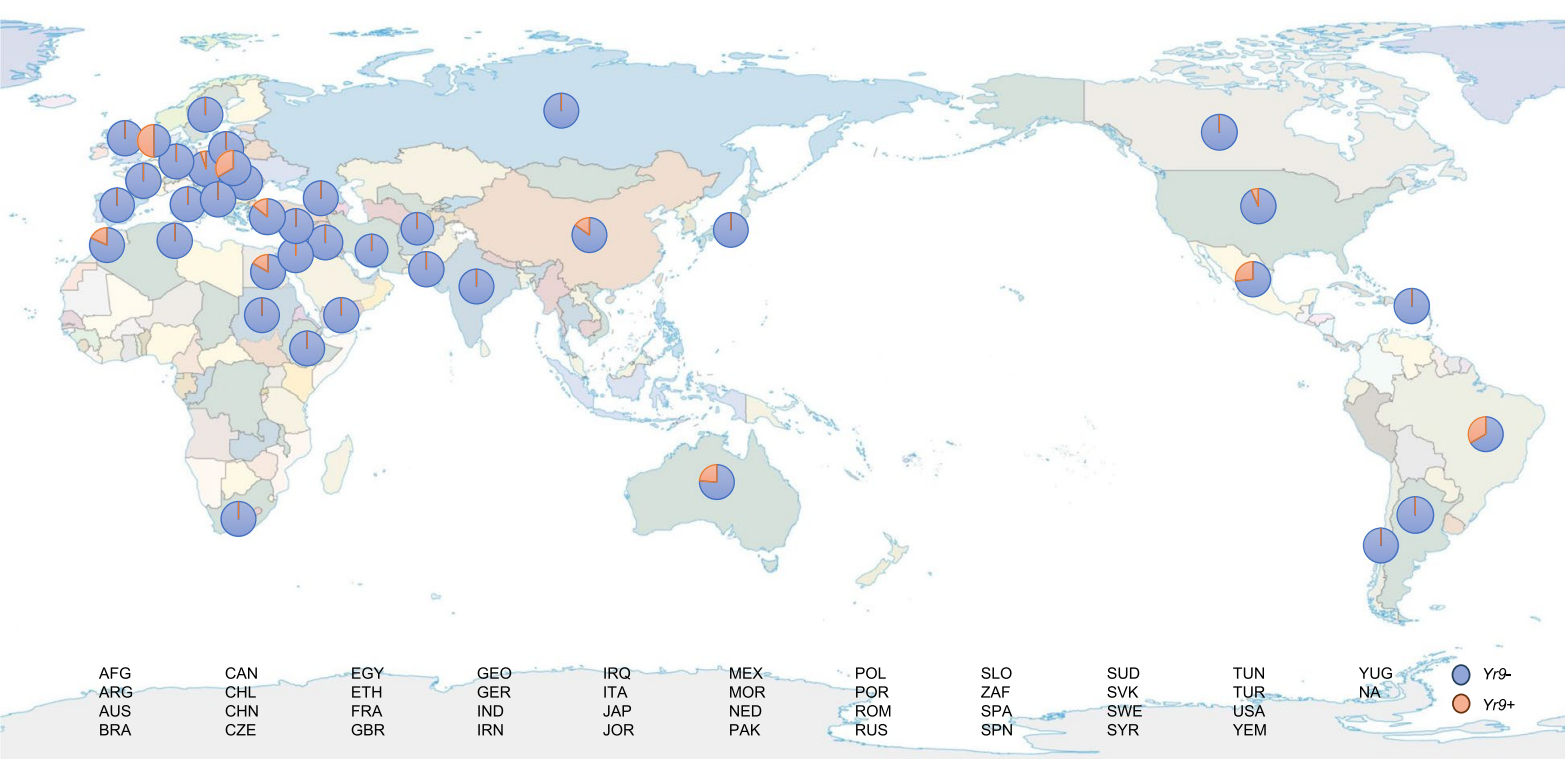
The graph of the frequency and distribution of *Yr9*. Using the developed *Yr9*-specific functional markers, several germplasms around the world were identified, and their frequency and distribution were counted. The orange area in the fan graph represents the presence of *Yr9*. The world map was downloaded at the website (http://bzdt.ch.mnr.gov.cn), and the map content approval number is GS(2016)1663

SNP loci *IWB103* and *IWB12298* were closely linked to *QYrxz.nwafu-2BL.5*. These two loci were developed as KASP molecular markers, and more than 300 modern Chinese wheat lines (varieties) were utilized for detection and genotyping. The results indicated that only the markers derived from *IWB12298* exhibited polymorphism across a wide range of varieties. Genotyping with the *IWB12298* marker predicted that 227 panel members possessed *QYrxz.nwafu-2BL.5*. Single-marker analyses confirmed that *IWB12298* was significantly associated with lower disease severity (DS) (*P* < 0.05). Accessions with alleles of *IWB12298* associated with *QYrxz.nwafu-2BL.5* had the lower DS scores (Table S10). Therefore, *IWB12298* were diagnostic for the presence of *QYrxz.nwafu-2BL.5* and can be employed in marker-assisted selection (MAS). Based on the *Yr9* gene sequence, we designed functional markers within the gene, with *Yr9M1* capable of distinguishing the presence of *Yr9* in a broad spectrum of collected germplasm. The carrier varieties of *Yr9*, such as Lovrin 10, Kenong 9204, and Aimengniu, exhibited stability in the marker tests. We used the developed molecular markers to assess the collected germplasms worldwide. Although the resistance conferred by *Yr9* is not as effective as it once was, 141 materials containing *Yr9* were identified in the tested germplasm, indicating that *Yr9* still exists in a considerable range of regions globally (Fig. 6).

## Discussion

We mapped *QYrxz.nwafu-1BL.6* on the long arm of chromosome 1B using IciMapping. It was located between the SNP markers *IWB71943* and *IWB5732*, within a 3.55 Mb interval (667.23–670.78 Mb). This location is near the multi-resistance gene *Yr29/Lr46/Sr58/Pm39*, which confer durable resistance to multiple diseases, especially stripe rust. Although this gene has not been cloned, some closely linked molecular markers, such as *csLV46G22*, has been developed (Cheng and Chen 2010). Genotyping wheat lines XZ9104 and AvS with the marker revealed that XZ9104 shares the same haplotype as positive controls Pavon 76, Attila, and Qinnong142, whereas AvS did not (Table S6). Cultivars with *Yr29* exhibit leaf tip necrosis in the field, as does XZ9104. Lines with *QYrxz.nwafu-1BL.6* alone showed slow rusting resistance, initially low disease severity and increasing at later growth stages. Therefore, we hypothesize that *QYrxz.nwafu-1BL.6* corresponds to *Yr29*. *Yr29* is common in cultivated varieties globally, which may facilitate its stability in the wheat breeding genome. However, its origin and impact on other agronomic traits are unclear and require further research.

*QYrxz.nwafu-3BS.7* is located between markers *IWB36792* and *IWB29059*, with a distance of 0.81 Mb. The *Yr30* gene was initially identified in the wheat variety Opata 85 and subsequently detected in other varieties, including Oligoculm, Pavon 76, and Chapio, among others (Singh et al. 2000; William et al. 2006; Yang et al. 2013; Suenaga et al. 2003). Besides conferring moderate resistance, this gene may be associated with gene controlling pseudo-black chaff (PBC) (Juliana et al. 2015). Cultivars with this gene may turn black in the glume shell and the lower internode area at the late filling stage, as do XZ9104. According to the previous fine mapping results of *Yr30*, we used the molecular markers *AX-95659840* and *AX-110053948* (Wang et al. 2024) co-segregated with this fine interval to genotype XZ9104 and AvS. The results showed that XZ9104 and Yaco 'S ' were grouped together, while AvS was in another group (Table S7). These results indicated that *QYrxz.nwafu-3BS.7* corresponds to *Yr30*.

*QYrxz.nwafu-2BL.5* accounted for 15.75–47.63% of the phenotypic variation, the largest effect among the three identified QTL. Currently, genes *Yr5, Yr7, Yr43, Yr44, Yr53*, and *Yr72* are located on chromosome 2BL (Marchal et al. 2018). *Yr5* confers resistance at all growth stages, and *Yr7* is an allele of *Yr5*. Additionally, *Yr43* (Cheng and Chen 2010), *Yr44* (Sui et al. 2009), *Yr53* (Xu et al. 2013), and *Yr72* (Chhetri et al. 2023) are also associated with ASR resistance. In contrast, XZ9104 shows resistance to CYR23 at the seedling stage due to the presence of *Yr9*, and *QYrxz.nwafu-2BL.5* has moderate resistance to multiple races in field, differing from the aforementioned genes. A total of 27 QTL have been mapped to chromosome 2BL, and a comparative analysis showed that the interval of *QYrxz.nwafu-2BL.5* nears to *QYr1* (Boukhatem et al. 2002) (Fig. S1). *QYrxz.nwafu-2BL.5* is situated within a hotspot region associated with other resistance QTL. Although it demonstrated an excepted effect on *Pst* resistance, comparative analysis revealed that it does not overlap with the QTL intervals identified by previous studies. However, the current evidence is inadequate to conclusively establish it as a novel QTL. AQP markers designed through tightly linked with *QYrxz.nwafu-2BL.5* can be used for molecular assisted selection breeding (Table S6). In this study, the combination of *Yr29*, *Yr30* and *QYrxz.nwafu-2BL.5* conferred durable resistance in the plants, and no significant antagonistic interactions were observed between these genetic factors. Furthermore, this finding offers a valuable reference for the pyramiding of disease resistance genes.

The wheat-rye 1RS.1BL translocation line, carries various disease resistance genes and increasing wheat yield, such as *Lr26*, *Sr31*, *Pm8*, *Gb2*, *Dn7*, *Yr9* (Hollenhorst and Joppa 1983; Mago et al. 2005; Marais et al. 1994; Mater et al. 2004; Tyler et al. 1987; Ren et al. 2022), is widely used in wheat breeding. Additionally, genes from rye, such as *Yr9* (Cai and Liu 1989), *Yr83* (Li et al. 2020), *Pm7* (Friebe et al. 1994), *Pm8* (Crespo-Herrera et al. 2017), *Pm17* (Hsam et al. 1994), and *Pm20* (Friebe et al. 1994), contribute to genetic diversity. *Yr9* has lost resistance to the current epidemic races due to extensive use Using the functional markers of *Yr9* for detection, *Yr9* still has a certain proportion in the global germplasm (Fig. 2; Table S8, S9). This may be due to the yield and disease resistance benefits of translocation lines. Although the effect of *Yr9* alone is less pronounced, its combination with other genes can enhance stripe rust resistance (Singh et al. 2022). Due to the advantageous traits conferred by varieties containing the *Yr9* gene, it still can be utilized in gene pyramiding strategies.

As the largest class of intracellular immune receptor family in plants, NLR genes can initiate the immune response at appropriate times to mitigate damage caused by pathogenic organisms. The structural characteristics of NLR genes are relatively conserved (Hui et al. 2022); however, during the course of evolution, these genes have continuously diversified through mechanisms such as replication, point mutations, and gene recombination to adapt to various biological stresses (Yang et al. 2008; Wicker et al. 2007). *Yr9* is a typical NLR gene, which exhibits a high degree of conservation, suggesting that it may share a common ancestral donor for resistance. Furthermore, analysis of the published KN9204 genome reveals that *Yr9* is situated within a resistance gene cluster, although the functions of the other resistance genes present in this cluster remain to be elucidated. The enrichment of cloned NLR (Nucleotide-binding site-Leucine-rich repeat) genes has enhanced our understanding of the recognition mechanisms employed by NLR proteins in response to various effectors. Concurrently, the recombination between NLR genes and other genes has the potential to further enrich the structure and function of NLR genes.

In this study, we combined map-based cloning with MutIsoSeq to clone the ASR gene *Yr9*. Unlike ASR genes which are easier to screen for mutants due to their higher effects and single-gene control, identifying mutants for APR genes that have lower effects poses challenges. Further exploration is needed for APR genes cloning. Map-based cloning accurately identifies resistance-conferring QTL, and mutant analysis quickly anchors candidate genes. The combination of these two approaches provides an effective methodology for the cloning of disease resistance genes.

## Materials and methods

### Plant materials

A population of 177 F_5_-derived F_6_ recombinant inbred line (RIL) was generated from a cross of susceptible Avocet S (AvS) and the resistant XingZi9104 (XZ9104). A panel of over 800 wheat cultivars, breeding lines, and Yr gene carriers were evaluated for response to stripe rust across multiple field environments and tested using known markers. The obtained data assessed the prevalence of resistance gene/QTL identified in XZ9104 based on flanking SNP markers. The wheat cultivars AvS, Mingxian 169 (MX169), and Xiaoyan 22 (XY22) served as susceptible controls.

### Field experiments

The RIL population derived from the XZ9104 and AvS was grown in six environments in random block design with two replications. The locations comprised Yangling in Shaanxi province (34°17’N, 108°04’E) during the 2014–2015 (14YL), 2015–2016 (15YL) and 2016–2017 (16YL), Jiangyou in Sichuan province (31°76’N, 104°72’E) in 2016–2017 (16JY), and Tianshui in Gansu province (34°89’N, 106°01’E) in 2015–2016 (15TS) and 2016–2017 (16TS). The location was selected according to suitability for infection. The cool and humid climate in Sichuan and southern Gansu provides an ideal environment for the natural infection and spread of stripe rust. At each site, 30 seeds of each line were sown in a 1-m single rows and the row spacing was 30 cm. A mixture of MX169 and XY22 was sown as susceptible spreaders after every 20 rows. In Yangling, the trials were inoculated with a mixture of the prevalent *Pst* races CYR32, CYR33 and CYR34, suspended in light oil (1:300), and sprayed onto MX169 and XY22 at flag leaf emergence. Phenotype of stripe rust at adult stage was recorded in Jiangyou (JY) in April, Yangling (YL) in May, and Tianshui (TS) in June, when severity of the susceptible control rows exceeded 80%.

### Greenhouse evaluation

To further validate the seedling resistance of XZ9104, multiple stripe rust races were utilized for inoculation and identification in the greenhouse. Twelve seeds of each test sample and control were planted in 15 × 15 × 15 cm plastic pots and held in a growth chamber conductive for seedling development. Sets of seedlings at the two-leaf stage, after which the corresponding *Pst* races of stripe rust were inoculated. The stripe rust and fluoride solution were mixed at a ratio of 1:200, thoroughly mixed, and inoculated with a pipette at 10 µl per leaf. After inoculation, a 24 h / 10 °C dark moisturizing treatment was performed, followed by transfer to a 16 h/16 °C – 8 h/10 °C environment for periodic light culture. The infection type of each line was recorded when the infection type (IT) on susceptible control MX169 reached 9.

### Phenotypic analysis

Analysis of variance (ANOVA) and Pearson’s correlation coefficients (r) were conducted using the “AOV” function in QTL IciMapping v4.2 software (Meng et al. 2015). Excel 2021 was used to calculate the frequency distribution of disease severity (DS) of the RIL population across 6 environments. Broad-sense heritability (h^2^) was estimated as h^2^ = σ^2^ g/(σ^2^ g + σ^2^ ge/e + σ^2^ ε/re), where σ^2^ g, σ^2^ ge and σ^2^ r represented genotypic (RILs), genotype (G) × environment (E), and error variances, σ^2^ g = (MSf – MSfe)/re, σ^2^ ge = (MSfe – MSe)/r, and σ^2^ e = MSe, MSf denoting the mean square of genotypes, MSfe the mean square of genotype–environment interaction, and MSe the mean square of error, and e and r were the numbers of environments and replicates. Additionally, the mean phenotypic values for all environments were employed to evaluate the genetic effects and identify the optimal confidence region for each QTL (Mu et al. 2019).

### Genotyping, linkage group construction and QTL mapping.

The genomic DNA of the population and parents was extracted by the CTAB method (Harbor 2009). Genotyping of RILs was performed using the wheat 90 K SNP array (http://www.molbreeding. com). The genotypes of the two parents were identified by the wheat 660 K SNP array (http://www.capitalbio.com). The separation of SNP markers was detected by χ^2^ goodness of fit test (*P* > 0.001), and markers whose segregation deviated from single locus expectation were excluded. Redundant and co-segregating markers were grouped using the 'BIN' function in QTL IciMapping v4.2 software (Meng et al. 2015). The selected markers were used to generate a genetic map using the 'MAP' function in IciMapping v4.2 software and visualized in Mapchart v2.3 (Voorrips 2022). The LOD significance thresholds were calculated with 1000 permutations at *P* ≤ 0.01, and LOD significance threshold used in estimates of each trait was 2.5. The Kosambi function (Kosambi 2016) was utilized to convert the recombination rate to centimorgans (cM). Phenotypic data for all environments including IT, DS and mean were used to identify QTL. Complete interval mapping was performed using the additive tool (ICIM-ADD) in IciMapping v4.2 to detect QTL. The phenotypic variation (PVE) explained by the additive effect at the peak of a single QTL and LOD was also obtained.

### Comparison with reported QTL and Yr genes

To establish the relationships between loci identified in this study and previously reported *Yr* genes and QTL, the flanking marker sequences associated with the previously reported *Yr* genes/QTL were used to obtain the physical positions in RefSeq v1.0 based on blast results (http://wheatomics.sdau.edu.cn/) (Ma et al. 2021). Based on SNPs from the 90 K SNP array genotypes of XZ9104 and AvS, KASP markers on chromosome arms 2BL were developed to detect *QYrxz.nwafu-2BL.5*. The PCR amplification procedure was described previously (Liu et al. 2022). Closely linked markers were used to determine the presence of *Yr29*, *Yr30* and other possible sites.

### Cytogenetic analysis

ND-FISH analysis of XZ9104 was conducted utilizing oligonucleotide probes. Oligo-pSc119.2, Oligo-pTa535, and Oligo-(GAA)7 were employed to identify rye and wheat chromatin, with the probe sequences referenced from Tang et al. (2014). Oligo-pTa535 was labeled with 6-TAMRA (6-Carboxytetramethylrhodamine) at the 5' end, while Oligo-pSc119.2 was labeled with 6-carboxyfluorescein at the 5' end. Additionally, Oligo-(GAA)7 was labeled with Cy5 (cyanidin 5) at the 5' end. The ND-FISH protocol was based on the method of Fu et al. (2015). Chromosomes were stained with 4',6-diamidino-2-phenylindole (DAPI). Detection of hybridization signals and image acquisition were performed using a Leica fluorescence microscope DM4B manufactured in Germany.

### EMS mutagenesis

A total of 2800 grains of wheat line XZ9104 with full and uniform grains were selected, washed with distilled water to remove impurities and dust, and evenly divided into multiple 1L reagent bottles. A preconfigured 50 mM sodium sulfate solution (500 ml) was added, and the seeds were soaked at a low temperature for 16 h. After draining the excess liquid, the seeds were incubated with 0.6% EMS solution on a shaker at 80 rpm for 16 h at room temperature. Post incubation, the seeds were placed in a 10% sodium thiosulfate solution for 1 h for the neutralization reaction. Subsequently, the seeds were then washed several times with distilled water, transferred to mesh bags, and rinsed under running water for over 1 h. After washing, the seeds were placed in a 50 × 30 cm black plastic tray with absorbent paper at the bottom, covered with a plastic lid, and vernalized at 4 °C for over 14 days. The vernalized grains were planted in a plug tray and placed in an artificial climate chamber with a 20 h day / 4 h night cycle and cultured at 22 °C / 18 °C until harvest. The harvested M_5_ plants were treated according to the above greenhouse inoculation method, and the susceptible mutant lines for the test were screened.

### Iso-seq library preparation and MutIsoSeq

CYR23 was used to inoculate Xingzi 9104 and the selected susceptible mutants at the two-leaf stage, and the leaves of the inoculated area at 0 h, 24 h, 48 h, 72 h, 96 h, and 7d were collected to obtain total RNA. The full-length transcriptome sequencing of the RNA sample of XZ9104 was performed using the PacBio Hifi sequencing, and the transcriptome sequencing of the susceptible mutant was conducted using the short reads sequencing. The library construction method and sequencing process are carried out as follow: A specific quantity of total RNA samples was used, and oligo dT beads were employed to enrich mRNA with poly A tail. The mRNA molecules were subsequently fragmented into small pieces. The fragmented mRNA was synthesized into first strand cDNA using random primers. The second strand cDNA was synthesized with dUTP instead of dTTP. The synthesized cDNA was subjected to end-repair and 3' adenylated. Adaptors were ligated to the ends of these 3' adenylated cDNA fragments. The U-labeled second-strand template was digested with Uracil-DNA-Glycosylase (UDG), and PCR amplification was performed. The library was amplified to produce DNA nanoballs (DNBs), which were sequenced using the DNBSEQ Technology platform. Using MutIsoSeq, the RNA-seq reads of the susceptible mutants were mapped to the reference sequence with the full-length transcript obtained by the Hifi sequencing as a reference, thereby identifying the transcripts carrying multiple independent EMS-type point mutations.

### Virus-induced gene silencing

The sequence obtained by Sanger sequencing was blasted against Wheat Omics 1.0 (http: //202.194.139.32/) and NCBI (https://blast.ncbi.nlm.nih.gov/Blast.cgi/), and detailed candidate gene information was obtained for known genes. Protein structure prediction is discussed in detail on the European Bioinformatics Institute's InterPro website (https://www.ebi.ac.uk/interpro/). To verify the function of the candidate gene, some low homologous sequences of the candidate gene CDS were selected as targets. Two pairs of target primers were used to amplify 170 bp (vigs1) and 187 bp (vigs2) fragments in the cDNA of XZ9104, respectively. The target fragment was inserted into the γ-pds vector by homologous recombination to obtain BSMV-γ(VIGS1) and BSMV-γ(VIGS2), thus completing the construction of the recombinant vector. The linearized recombinant vector and the original viral vector ( BSMV-α, BSMV-β, BSMV-γ) were transcribed in vitro. The transcription products were mixed in equal volume to form a recombinant virus, which was mixed with FES buffer to form a mixture (1.5 μL / 8.5 μL) and inoculated into wheat leaves. After 10 days of culture at 25 ℃, when the symptoms were visible, the plants were inoculated with the *Pst* race CYR23 of stripe rust according to the above method. The symptoms of the disease were evaluated 14 days post inoculation.

## Conclusion

This study identified three stable APR QTL (*QYrxz.nwafu-1BL.6/Yr29*, *QYrxz.nwafu-3BS.7/Yr30*, and *QYrxz.nwafu-2BL.5*) and cloned the ASR gene *Yr9* with a CC-NBS-LRR structure from XZ9104. These genes make XZ9104 a valuable breeding parent. The developed molecular markers will enhance MAS for breeding durable disease-resistant wheat varieties. Future work should focus on fine mapping *QYrxz.nwafu-2BL.5* and further characterizing these genes to improve our understanding of wheat disease resistance mechanisms.

## Supplementary Information

Supplementary Material 1. Table S1 Genetic analysis of resistance to CYR23 at seedling stage. Table S2 Distribution of single-nucleotide polymorphism (SNP) markers on 21 wheat chromosomes in genetic map by AvS × XZ9104 RIL population. Table S3 Summary of stripe rust resistance QTL detected in the AvS × XZ9104 RIL population in the seedling using IciMapping V4.2. Table S4 Effects of different QTL combinations in the RILs from AvS × XZ9104 population based on DS data in five field experiments (Yangling, Tianshui, and Jiangyou during the 2014-2016 cropping seasons). Table S5 Mutations in *YrXZ* induced by EMS mutagenesis. Table S6 Allele-specific quantitative PCR (AQP) primers used for marker-assisted breeding *QYrxz.nwafu-2BL.5*. Table S7 Molecular markers used for known gene detection. Table S8 The 816 accessions used in validation of *Yr9* functional markers. Table S9 Sequence of Gene-specific functional molecular markers of*Yr9*. Table S10 The 366 accessions used in validation of marker *IWB12298.*

Supplementary Material 2. Fig S1 The physical location of *QYrxz.nwafu-2BL.5 *and reported genes/QTL that marked in red and blue, respectively. Fig S2 Changes in the amino acid sequence encoded in the *Yr9* susceptible mutants(https://www.novopro.cn/tools/muscle.html). Fig S3 The amplification of *Yr9* functional markers in different materials. Fig S4 Marker for *QYrxz.nwafu-2BL.5*. Single marker analysis of KASP markers *IWB12298* in 366 Chinese lines/cultivars. *QYrxz.nwafu-2BL.5*+, accessions with *QYrxz.nwafu-2BL.5*; *QYrxz.nwafu-2BL.5-*, accessions lacking *QYrxz.nwafu-2BL.5*. Black, red and blue dots, representing NTC, HEX and FAM, respectively. Fig S5 (a) Virus induced gene silencing (VIGS) test of the *YrXZ* gene on the Avocet+*Yr9* line. (b) After inoculation with CYR23, the biomass of *Pst* in the silenced leaves of *YrXZ* was measured by *PstEF* and *TaEF *as internal reference genes. The values are presented as the mean ±SD (n=3). A t-test was employed for significance analysis (***P<0.001). Fig S6 Evolutionary tree between *Yr9* and cloned NLR genes in crops.

## Data Availability

The datasets mentioned in this study are available in a published article and various online repositories. Details regarding the repository/repositories and corresponding accession number(s) can be found in the Supplementary Material section.

## Abbreviations

ASR: All-stage resistance
APR: Adult-plant resistance
ANOVA: Analysis of variance
ADD: Additive
cM: CentiMorgans
DS: Disease severity
EMS: Ethylmethane sulfonate
ICIM: Inclusive composite interval mapping
KASP: Kompetitive allele-specific PCR
MAS: Marker-assisted selection
QTL: Quantitative trait locus
RILs: Recombinant inbred lines
SNP: Single nucleotide polymorphism
LOD: Logarithm of odds
VIGS: Virus-induced gene silencing
*Pst*: *Puccinia striiformis *f. sp.* Tritici*
