# Supplementary material for: Rapid isolation of Yr9 via MutIsoSeq and QTL analysis of durable stripe rust resistance in wheat cultivar Xingzi 9104
Source: Stress Biol. 2025 Apr 30;5(1):29. doi: 10.1007/s44154-025-00226-9 (PMC12044127; doi:10.1007/s44154-025-00226-9)
Supplement: Supplementary file 2 — Supplementary Material 2. Fig S1 The physical location of QYrxz.nwafu-2BL.5 and reported genes/QTL that marked in red and blue, respectively. Fig S2 Changes in the amino acid sequence encoded in the Yr9 susceptible mutants(https://www.novopro.cn/tools/muscle.html). Fig S3 The amplification of Yr9 functional markers in different materials. Fig S4 Marker for QYrxz.nwafu-2BL.5. Single marker analysis of KASP markers IWB12298 in 366 Chinese lines/cultivars. QYrxz.nwafu-2BL.5+, accessions with QYrxz.nwafu-2BL.5; QYrxz.nwafu-2BL.5-, accessions lacking QYrxz.nwafu-2BL.5. Black, red and blue dots, representing NTC, HEX and FAM, respectively. Fig S5 (a) Virus induced gene silencing (VIGS) test of the YrXZ gene on the Avocet+Yr9 line. (b) After inoculation with CYR23, the biomass of Pst in the silenced leaves of YrXZ was measured by PstEF and TaEF as internal reference genes. The values are presented as the mean ±SD (n=3). A t-test was employed for significance analysis (***P<0.001). Fig S6 Evolutionary tree between Yr9 and cloned NLR genes in crops. [file 44154_2025_226_MOESM2_ESM.pptx]

## Slide 1
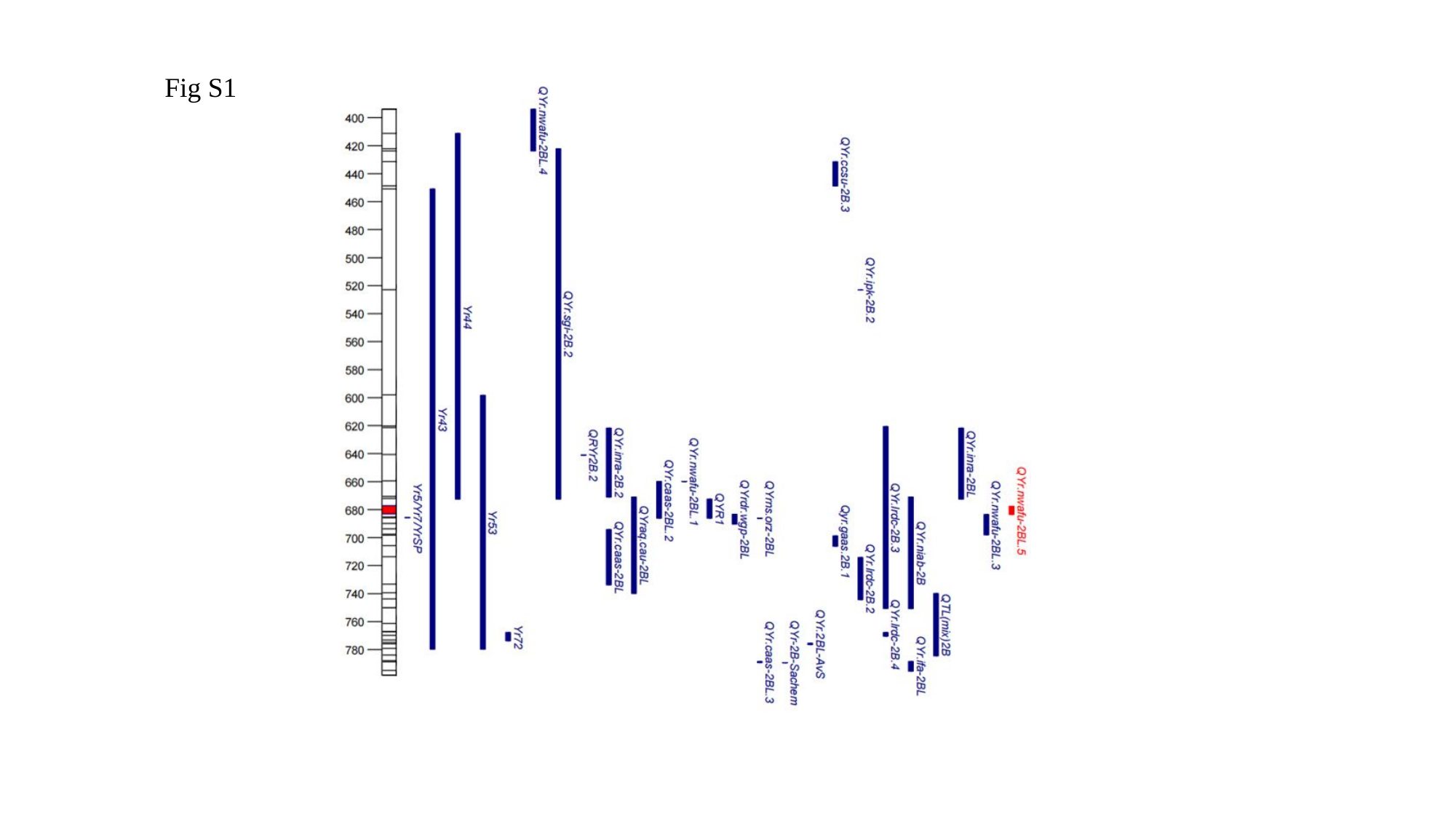

Fig S1

## Slide 2
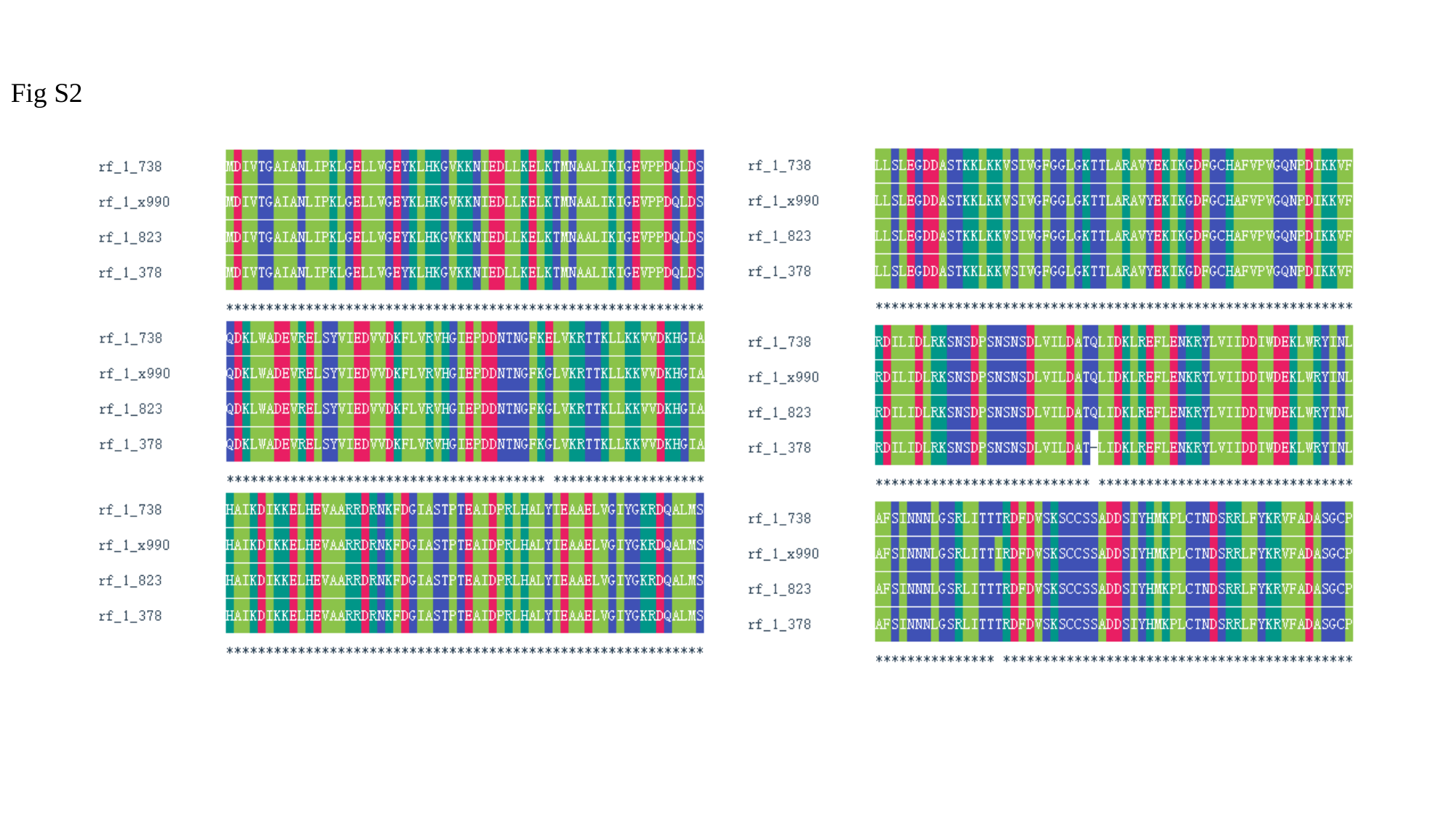

Fig S2

## Slide 3
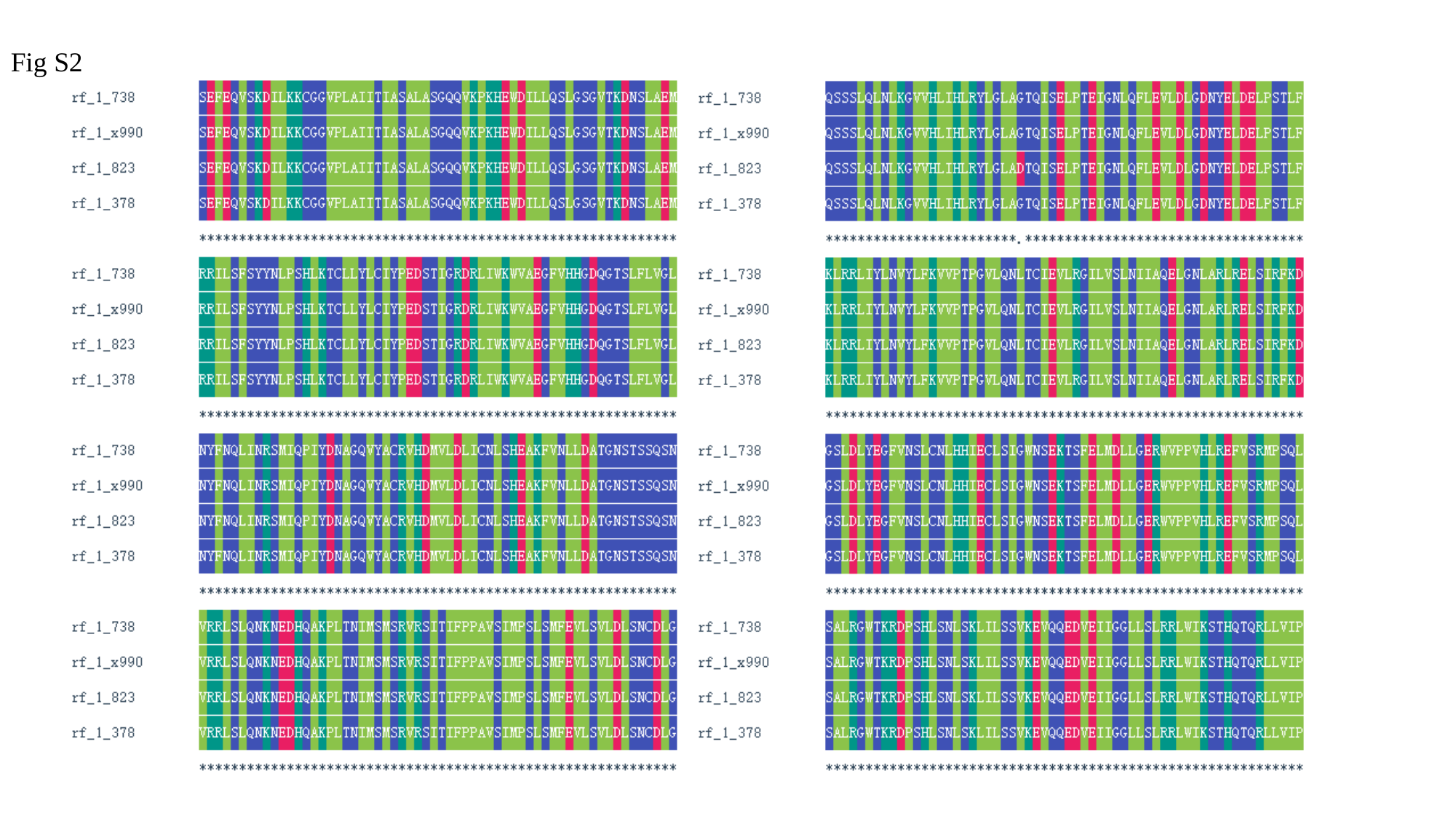

Fig S2

## Slide 4
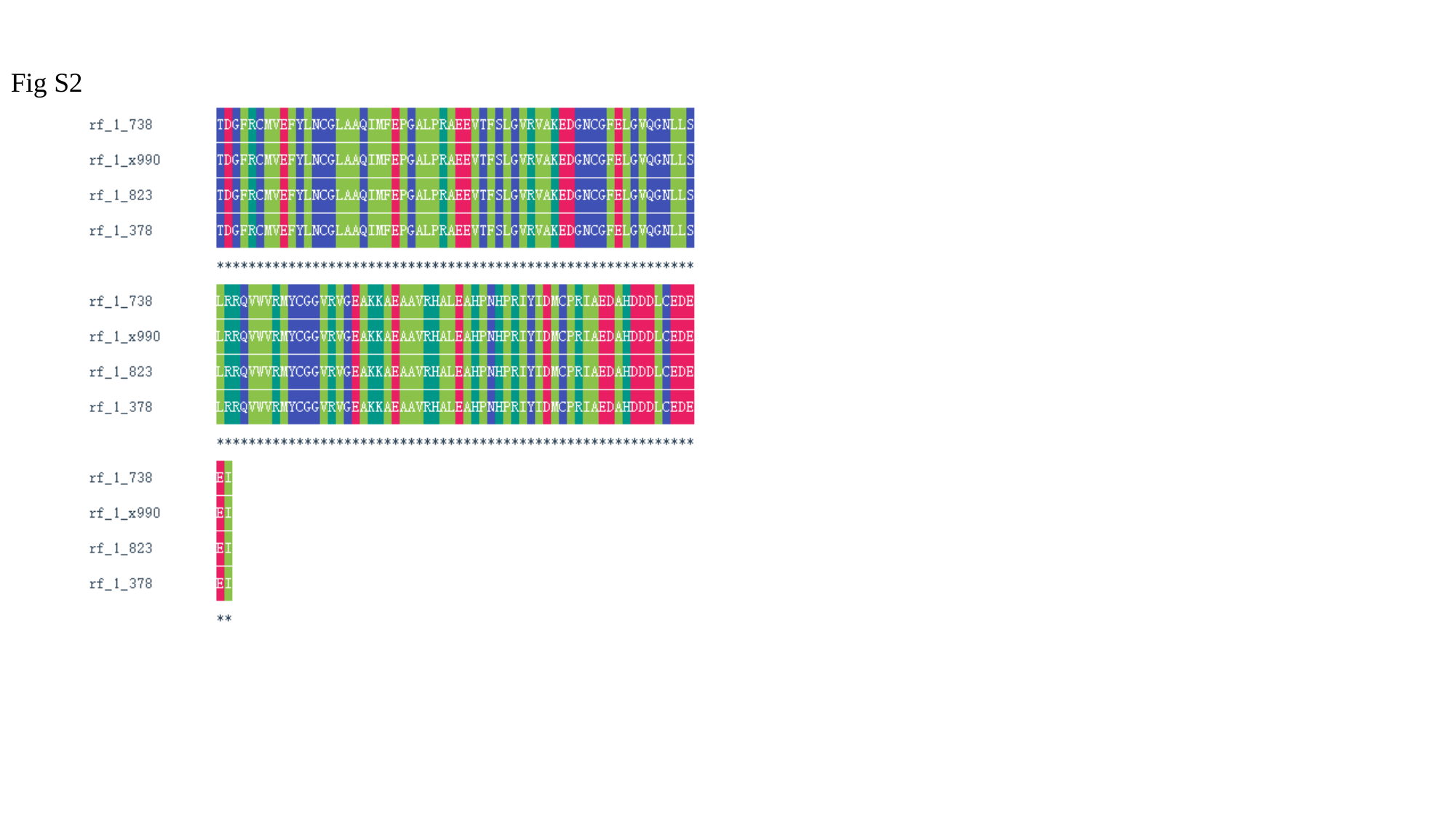

Fig S2

## Slide 5
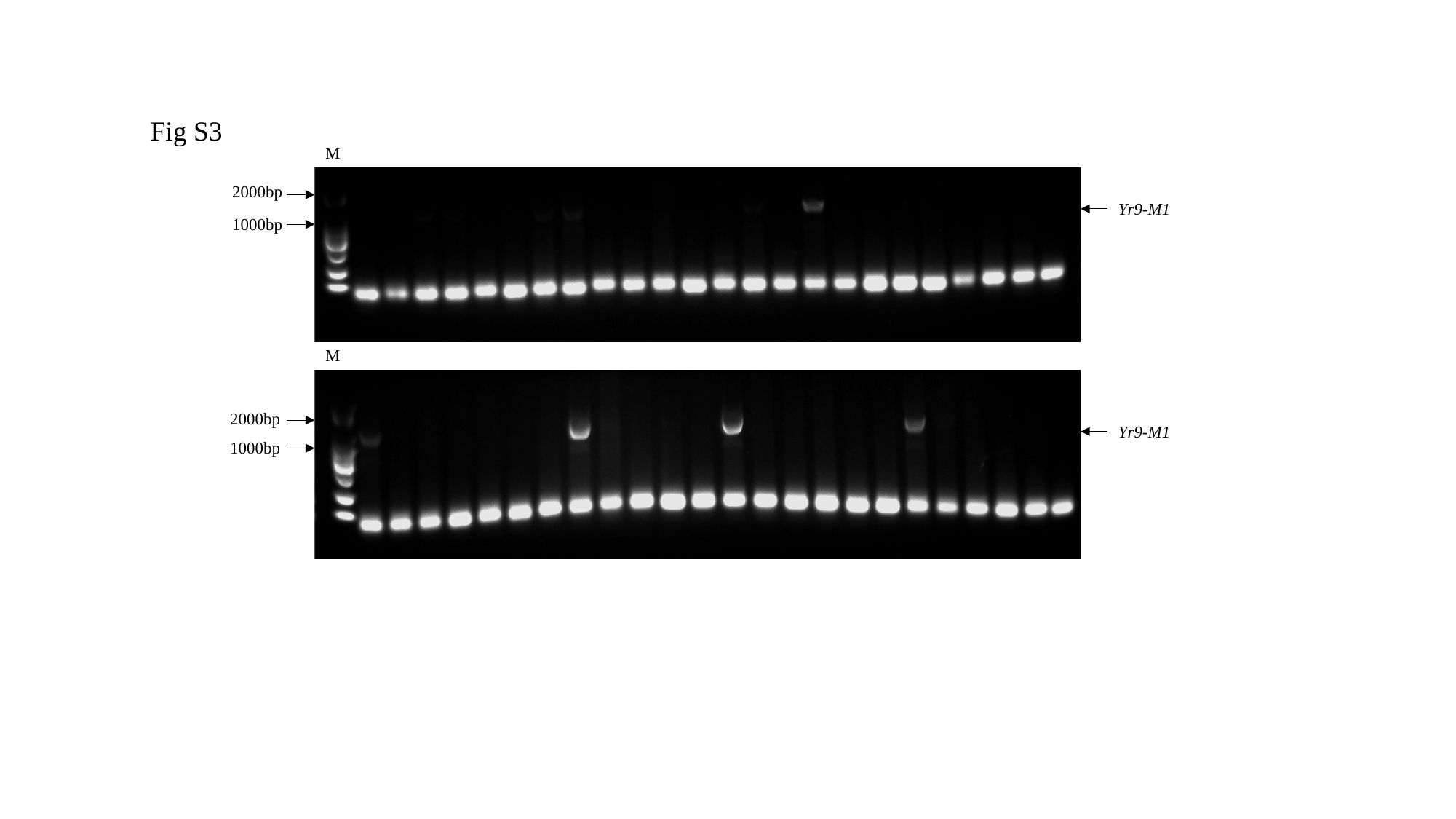

Fig S3
M
Yr9-M1
M
Yr9-M1
2000bp
1000bp
2000bp
1000bp

## Slide 6
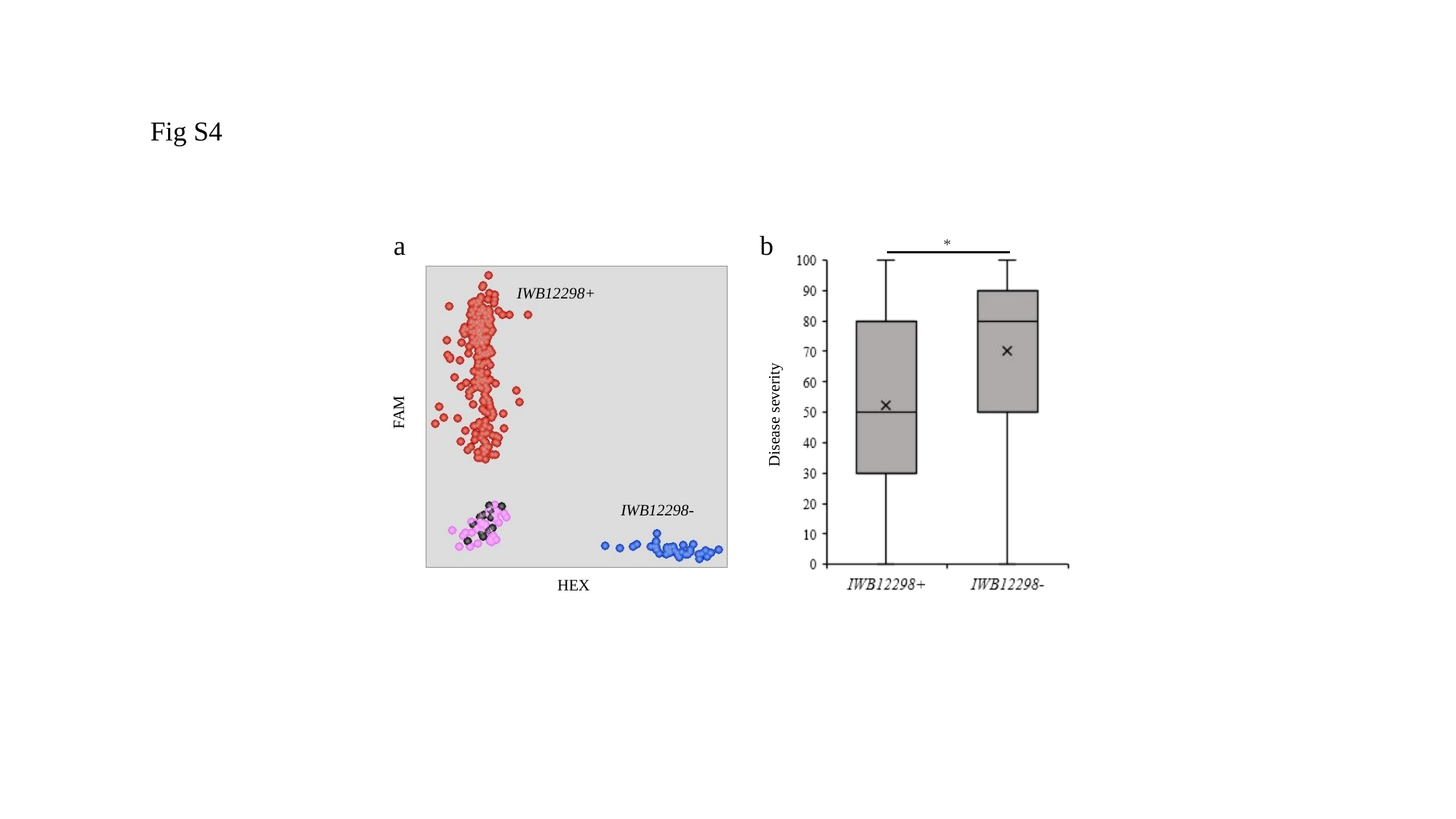

Fig S4
a
b
IWB12298+
FAM
Disease severity
IWB12298-
HEX
*

## Slide 7
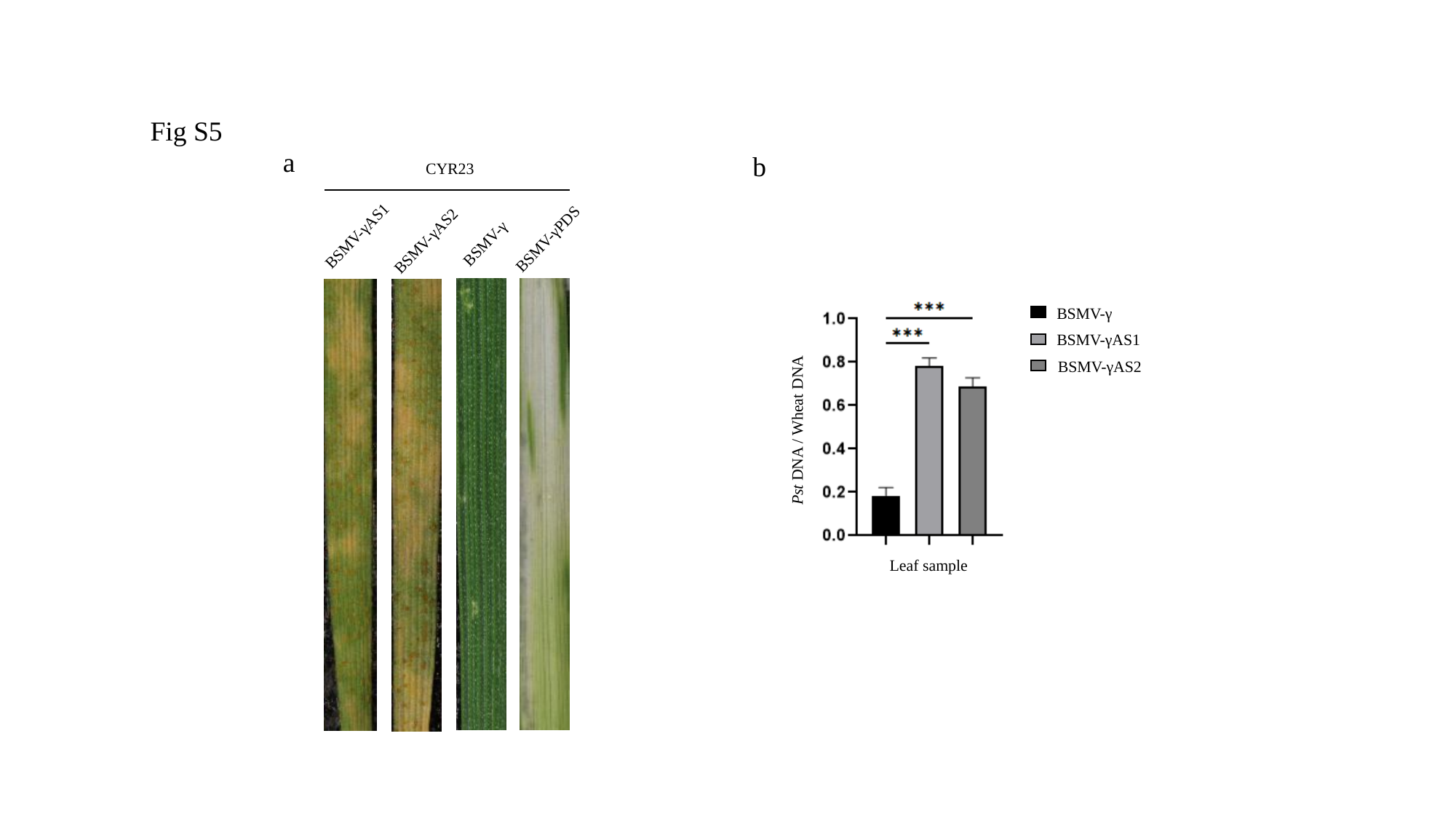

Fig S5
a
b
CYR23
BSMV-γAS1
BSMV-γPDS
BSMV-γAS2
BSMV-γ
BSMV-γ
BSMV-γAS1
BSMV-γAS2
Pst DNA / Wheat DNA
Leaf sample

## Slide 8
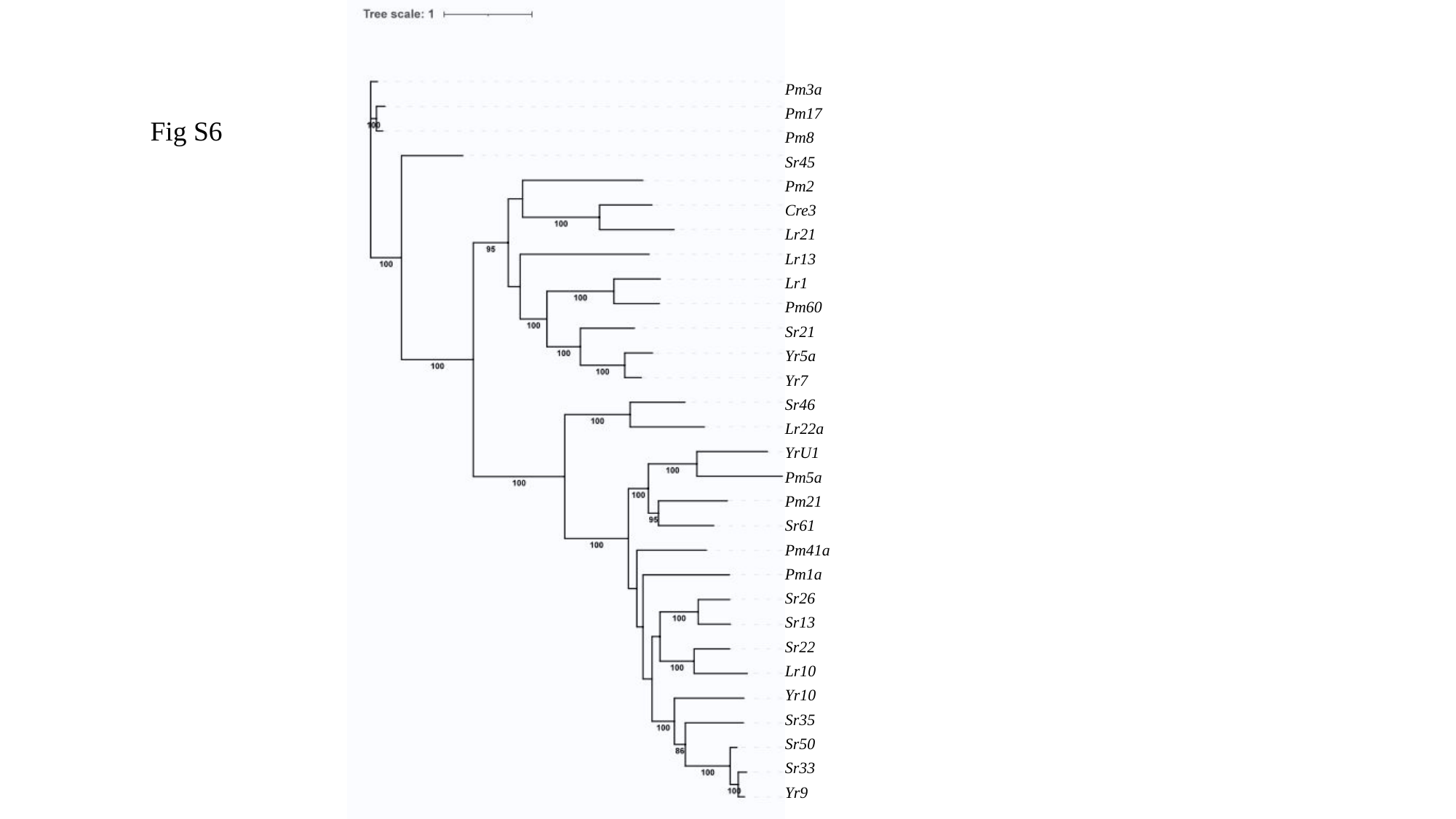

Pm3a
Pm17
Pm8
Sr45
Pm2
Cre3
Lr21
Lr13
Lr1
Pm60
Sr21
Yr5a
Yr7
Sr46
Lr22a
YrU1
Pm5a
Pm21
Sr61
Pm41a
Pm1a
Sr26
Sr13
Sr22
Lr10
Yr10
Sr35
Sr50
Sr33
Yr9
Fig S6
